# Design and Fabrication of Piezoelectric Micromachined Ultrasound Transducer (pMUT) with Partially-Etched ZnO Film

**DOI:** 10.3390/s17061381

**Published:** 2017-06-14

**Authors:** Junhong Li, Wei Ren, Guoxiang Fan, Chenghao Wang

**Affiliations:** State Key Laboratory of Acoustics, Institute of Acoustics, Chinese Academy of Science, Beijing 100190, China; renwei@mail.ioa.ac.cn (W.R.); fanguoxiang13@mails.ucas.ac.cn (G.F.); chwang@mail.ioa.ac.cn (C.W.)

**Keywords:** pMUT, centric electrode structure, ZnO film, partially-etched, sensitivity

## Abstract

A square piezoelectric composite diaphragm was analyzed by the finite element method to enhance the sensitivity of a piezoelectric micromachined ultrasound transducer (pMUT). The structures of electrode and piezoelectric film were optimized and a centric electrode was designed to avoid the counteraction of stress in the centre and edges. In order to further improve the sensitivity; a pMUT with partially-etched piezoelectric film was adopted. The receive and transmit sensitivities of the pMUT were analyzed in details. The receive sensitivity of pMUT with partially-etched ZnO film is 3.3 dB or 6.8 dB higher than those with a centric and whole electrode, respectively; and the amplitude of a partially-etched ZnO film pMUT under a certain voltage is 5.5 dB and 30 dB higher than those with centric and whole electrode separately. Two pMUT-based ZnO films were fabricated by micromachining technology and their receive and transmit sensitivities were tested. The ZnO films deposited by direct current (DC) magnetron sputtering exhibit a densely packed structure with columnar crystallites. The test results show that the structure of the square diaphragm with partially-etched piezoelectric layer can significantly improve the transducer sensitivity. The receive sensitivity and transmit sensitivity are −238.35 dB (ref. 1 V/μPa) and 150.42 dB (ref. 1 μPa/V); respectively.

## 1. Introduction

**Figure 1:**
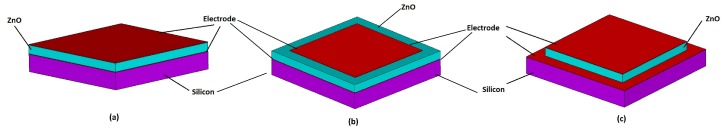
Schematic structure of pMUT’s vibrating diaphragm with (**a**) whole electrode; (**b**) centric electrode and (**c**) partially-etching ZnO film.

Micromachined ultrasound transducers (MUTs) have been extensively developed. Arrays with high element density, small element size and high resonant frequency are easy to realize by MUT [1,2,3]. Moreover, the MUT array and external circuit can be integrated in a chip. Therefore, MUT arrays have wide applications in medical acoustic imaging, ultrasonic fingerprint identification and ultrasonic vein identification [4,5,6,7,8,9,10]. The MUTs work on either electrostatic or piezoelectric principles, and they can be thus be classified as capacitive MUTs (cMUTs) and piezoelectric MUTs (pMUTs). Compared with cMUTs, pMUTs have lower impedance, which may allow more energy input and can avoid the DC-biased voltage cMUTs need [11,12]. The pMUT usually works in bending mode, and a few in extensional mode and d_33_ mode [13,14]. The properties of a MUT array depend greatly on the sensitivity of the single element transducers. The piezoelectric film and structure of pMUT are two important factors that affect the sensitivity. ZnO and PZT films are usually used as piezoelectric layers in pMUTs [15,16,17,18,19,20]. Compared with that of PZT film the deposition process of ZnO film is simple and compatible with micromachining technology [21,22,23,24,25,26,27]. In order to improve the properties of pMUT, Belgacem studied the influence of centred and ring top electrode on the coupling factor of pMUT with a circular piezoelectric composite diaphragm released by the expensive deep reactive ion etch (DRIE) process, and they found that when the PZT was removed outside the electrode this can improve the coupling factor [28]. The vibration diaphragms of pMUTs include piezoelectric films and non-piezoelectric layers, and if released by wet etching they are square for anisotropic etching of silicon [29,30,31]. The wet etching process is simpler and cheaper than DRIE that can realize circular piezoelectric composite diaphragm. The theory analysis of square piezoelectric composite diaphragms is difficult to be carried out by analytical methods. The working process of pMUTs involves the transmission and reception of sound waves, thus the receive and transmit sensitivity of pMUTs need to be analyzed. In our work, a ZnO film was deposited by direct current (DC) magnetron sputtering and used as a piezoelectric material in a pMUT. As shown in Figure 1, the sensitivities of three kinds of pMUTs, including whole electrode, centric electrode and partially-etched ZnO film were analyzed by the finite element method (FEM), and a centric electrode and a partially-etched ZnO film were adopted to improve the sensitivity. Two pMUTs were designed and fabricated by a micromachining process. Finally, the sensitivities of pMUTs with partially-etched and whole ZnO films were tested and compared.

## 2. Finite Element Analysis for a Piezoelectric Composite Diaphragm

**Figure 2:**
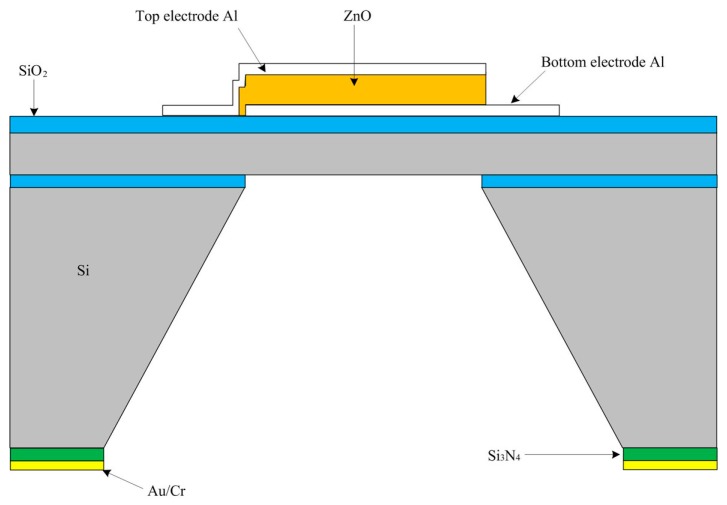
Schematic structure of the piezoelectric micromachined ultrasound transducer.

The pMUT needs to work at a higher resonant frequency for an ultrasound imaging system with higher resolution, and the resonant frequency depends heavily on the thickness of the vibrating diaphragm. Silicon-on-insulator (SOI) wafers have accurate silicon layer thicknesses which usually ranges from several to dozens of microns, so they are suitable for pMUTs [32,33,34]. The control of bulk silicon etching time is another method to get a certain silicon layer thickness [2,14,30]. As shown in Figure 2, the piezoelectric composite vibrating diaphragm of a pMUT includes a non-piezoelectric vibration membrane and a piezoelectric film with top and bottom electrodes.

The resonant frequency of a pMUT is proportional to the thickness of the vibration diaphragm and piezoelectric film. Therefore, the thickness of the vibrating membrane is usually greater than 5 μm. The resonant frequency and sensitivities of three kinds of pMUTs, including whole electrode, centric electrode and partially-etched ZnO films, were analyzed by the finite element method (FEM). The schematic structures of the pMUT’s vibrating diaphragm with whole electrode, centric electrode and partially-etching ZnO film are shown in Figure 1. In finite element analysis, the up and bottom electrodes are 0.2 μm thick Al, and a 3 μm thick ZnO film is used as the piezoelectric layer. The piezoelectric composite vibrating diaphragm is square and 426 μm wide. The non-piezoelectric vibration diaphragm comprises 0.2 μm thick oxide and a 6 μm thick silicon device layer. The substrate silicon is 300 μm thick and anisotropically wet etched to release the vibrating diaphragm. In our work, the modal and static analysis of pMUT were studied by means of FEM using ANSYS. The eight-node hexahedral coupled-field element SOLID5 and the eight-node linear structural element SOLID45 were used for ZnO films and silicon, respectively. The structure parameters and properties of materials used in the FEM are listed in Table 1.

In our FEM simulation, the effect of structural damping was neglected and the displacement degrees of freedom were constrained to be zero as the fixed four lateral faces of the piezoelectric ZnO/SiO_2_/Si composite diaphragm that can be regarded as square thin plate. The vibration equation of a square composite thin plate can be written as:

(1) $$\frac{\partial^{4}w}{\partial x^{4}}+2\frac{\partial^{2}w}{\partial x^{2}\partial y^{2}}+\frac{\partial^{4}w}{\partial y^{4}}+\frac{\partial^{2}w}{\partial t^{2}}\frac{\rho h}{D}=0$$

where *w* is the displacement, *D* is the bending rigidity, *ρ* and *h* are the average density and thickness of the composite diaphragm, respectively.

**Figure 3:**
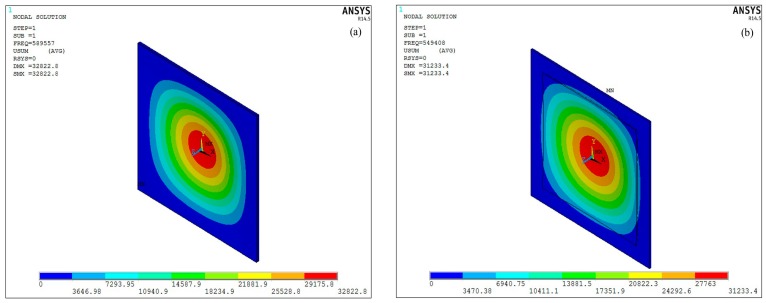
The displacement distributes of the piezoelectric composite diaphragm at first resonance mode (**a**) for whole or centric electrode and (**b**) for partially-etching ZnO film.

The modal analysis of the piezoelectric composite diaphragm was carried out to predict the resonant frequency, as shown in Figure 3. The resonant frequency of pMUT with whole and centric electrode are around 590 KHz, that is approximately equal to that calculated by the following expression [35]:

(2) $$f_{11}=\frac{17.994}{\pi a^{2}}\sqrt{\frac{D}{\rho h}}$$

where *D* is the bending rigidity , *ρ*, *a* and *h* are is average density, length and thickness of the composite diaphragm respectively. 

From the above expression, we can see that the resonant frequency of pMUT is proportional to thickness and inversely proportional to the width, respectively, which is in agreement with the results of other’s research [2,14]. As shown in Figure 3b, the resonant frequency of a pMUT with partially-etching ZnO film is about 93% of that with a whole piezoelectric film.

**Figure 4:**
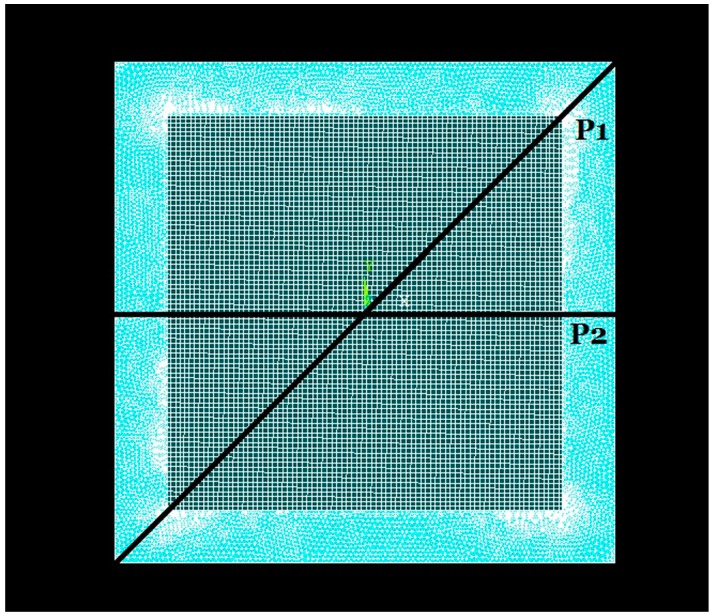
The finite element mesh of the composite diaphragm.

**Figure 5:**
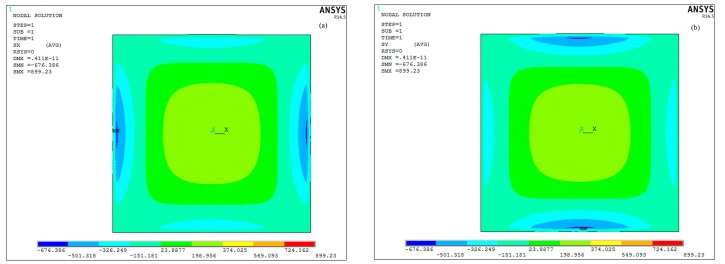
The stress distributions of the piezoelectric composite diaphragm in (**a**) the X and (**b**) the Y direction.

**Figure 6:**
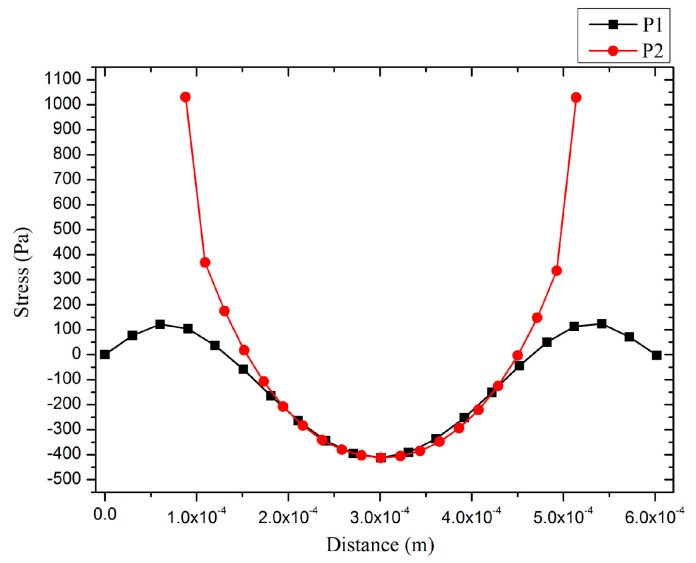
The stresses distributions in piezoelectric film of pMUT with whole electrode.

The receive sensitivity *S* of pMUT can be calculated as [36]:

(3) $$S=\frac{d_{31}}{\varepsilon AP}\int_{0}^{a}\int_{0}^{a}h\sigma dxdy$$

where *P* is the sound pressure on the pMUT, *A* and *a* are the area and length of the composite diaphragm separately, and *ε*, *d*_31_, *h*, *σ* are the permittivity, piezoelectric (strain) coefficient , thickness and stress of the piezoelectric films, respectively. Due to the other parameters being equal, a greater stress means a higher receive sensitivity. To obtain the stress distribution in the piezoelectric composite diaphragms under uniform pressure load, a uniform pressure load (1 Pa) was imposed on composite diaphragm in FEM static analyses. As shown in Figure 4, the stress and displacement analyses were carried out along line P1 and P2. Figure 5 and Figure 6 show the stresses distributions of pMUT with whole piezoelectric film. The stresses from edge to centric change gradually. The stresses in the centre and edge are bigger than in other areas, but they are opposite. Therefore, the partial stress will be offset if the electrodes cover the entire piezoelectric film, and the sensitivity of the pMUT will decrease.

**Figure 7:**
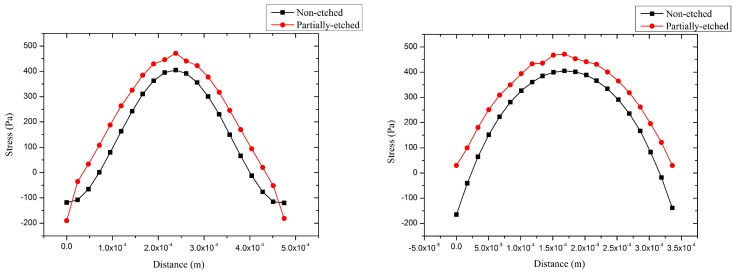
The stresses distributions in piezoelectric film of pMUT with whole and partially-etched ZnO films along P1 (**left**) and P2 (**right**) lines.

Figure 7 shows the stress distributions in the piezoelectric film with a centric electrode and partially-etched ZnO film. The average stress of the composite vibration diaphragm with centric electrode is higher by around 3.5 dB than those with a whole electrode by avoiding the counteraction of stress between the centre and out-ring regions. At the same time, the fact that ZnO film in the outer-ring region was etched leads to a further increase of the average stress in the piezoelectric film. The stress with partially-etching ZnO film is higher by around 3.3 dB and 6.8 dB than those with centric and whole electrodes, respectively, which means the receive sensitivity will increase by 3.3 dB and 6.8 dB.

**Figure 8:**
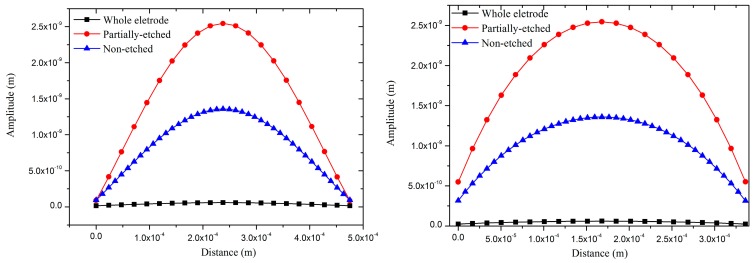
The amplitudes of pMUT with whole electrode, centric electrode and partially-etched ZnO films along P1 (**left**) and P2 (**right**) lines.

The transmit sensitivity of pMUT is proportional to the amplitude of the vibration diaphragm [30]. The amplitudes of pMUTs with whole, centric electrode and partially-etched ZnO films were compared by FEM static analysis, and a 1 V voltage was loaded on the piezoelectric film in the analyses. Figure 8 shows the amplitudes of pMUTs with whole electrode, centric electrode and partially-etching ZnO film. The amplitude of the pMUT with whole electrode is much lower than those with centric electrode and partially-etching ZnO film due to the offset of stress between the centric and out-ring regions. The amplitude of the pMUT with partially-etched ZnO film is higher by 5.5 dB than that with a whole piezoelectric film, which can be attributed to the change of boundary conditions of the vibrating diaphragm. In our work, two pMUTs with centric electrode and partially-etched ZnO film were fabricated, and their transmit and receive sensitivities were tested and compared.

## 3. Microfabrication of pMUT

**Figure 9:**
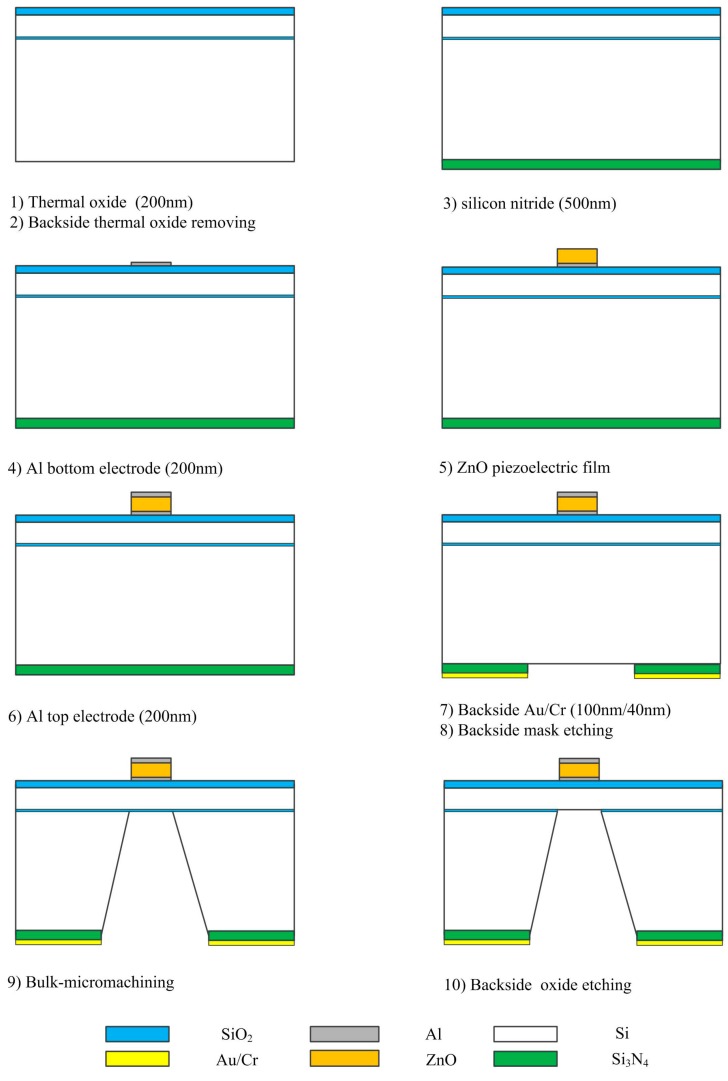
Fabrication process of the pMUT.

The pMUT has been fabricated using standard bulk micromachining fabrication techniques. Silicon nitride, silicon oxide and polysilicon are usually used as vibration diaphragms of MEMS devices, but the thicknesses of the three films are not more than 1 μm due to the deposition technique limits [37,38,39,40]. To get a higher and more accurate resonant frequency, the vibration diaphragm of pMUT should have greater and more precise thickness. Therefore, a layer of silicon got by controlling of bulk silicon etching time or SOI wafer was used as the vibration membrane of the pMUT (shown in Figure 9 is SOI wafer). The diaphragm was released by silicon anisotropic wet etching. The silicon anisotropic wet etching mask must be compact and anticorrosive to safeguard the wafer and successfully finish the pMUT microfabrication. On the basis of process experiments, a composite of Si_3_N_4_ deposited by plasma enhanced chemical vapor deposition (PECVD) and Au/Cr film was chosen as the mask.

**Figure 10:**
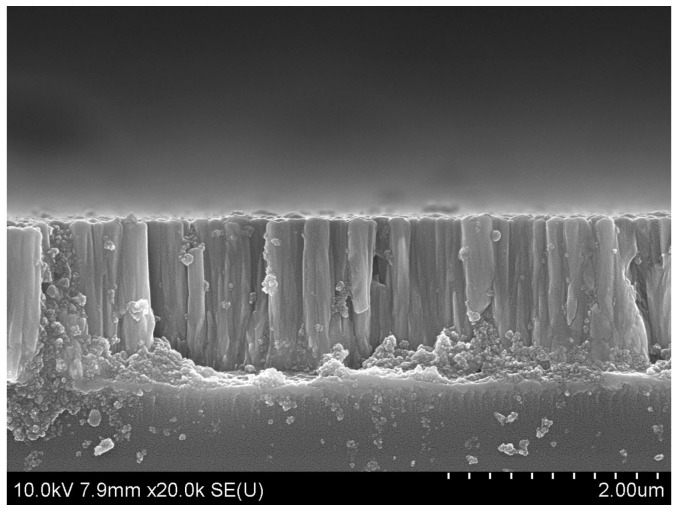
SEM picture of the cross-section view of the ZnO films.

A description of the pMUT microfabrication is shown in Figure 9. The wafers were firstly wet oxidized at 1100 °C to grow a 0.2 μm thick thermal oxide layer, which was removed from the back side of the wafer using hydrofluoric acid solution. A 0.5 μm thick silicon nitride film was then deposited by PECVD on the back side of the wafer to be used as the mask for the silicon anisotropic wet etching, followed by a 0.2 μm thick Al film deposited by ion-beam sputtering and patterned by standard photolithography techniques and wet etching in H_3_PO_4_ solution to form the bottom electrode. After that ZnO films were deposited on the bottom electrode, and patterned by a wet etching process using H_3_PO_4_ solution to finish the preparation of the piezoelectric layer. The effects of Ar/O_2_ ratio and sputtering pressure on the properties of ZnO films deposited using dc magnetron sputtering were investigated, and the preparation parameters were optimized. As shown in Figure 10, the ZnO film exhibits a densely packed structure with columnar crystallites preferentially oriented along the (002) plane, which indicates the film possesses good piezoelectric properties. Following the preparation and patterning of the piezoelectric layer, a 0.2 μm thick Al film was deposited by ion-beam sputtering and photolithographically patterned by lift-off processing to form the top electrode. The Au/Cr film was then deposited on the back side of wafer and patterned by back-to-front alignment photolithography techniques and a wet etching process, followed by inductively coupled plasma dry etching of PECVD silicon nitride to form the mask for silicon wet etching. Thereafter, the wafer was fixed in a Teflon chucking appliance, and was anisotropically etched using a KOH etchant at 70 °C to release the diaphragm, The bulk silicon was wet etched until the required thickness was achieved, then the oxide was removed by hydrofluoric acid solution from the diaphragm. Finally, the wafer was unloaded from the fixture, and washed with deionized water.

## 4. Results and Discussion

**Figure 11:**
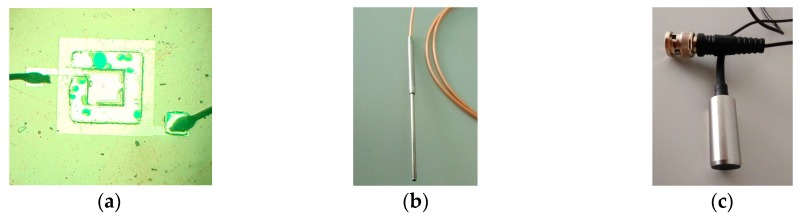
Optical images of (**a**) pMUT with partially-etched ZnO film and the calibrated (**b**) hydrophone; (**c**) transmit transducer used in sensitivity test.

The pMUTs with whole and partially-etched ZnO film (the width of the square diaphragm is 470 μm, the thickness of ZnO, silicon oxide and silicon layers are 4, 0.2 and 33 μm, respectively) were fabricated by the above micromachining technique. As shown in Figure 11a, the ZnO films was partially etched, and the bottom electrode was also slightly etched during the process of removing of piezoelectric film. To measure the properties of devices, the pMUT dies were glued on a printed circuit board (PCB), electrically connected by ultrasonic pressure welding and packaged in metal box with a window where the vibration diaphragm of pMUT was exposed.

**Figure 12:**
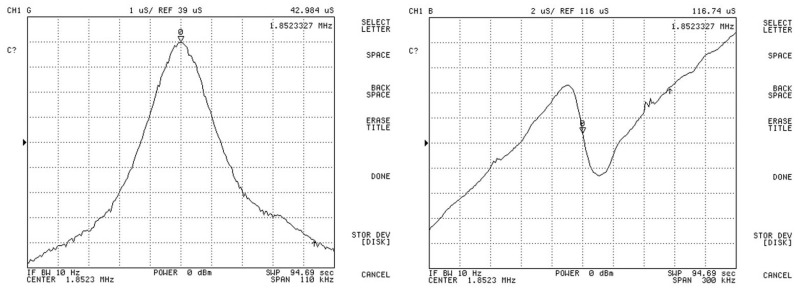
The admittance curves of pMUT with partially-etched ZnO film.

**Figure 13:**
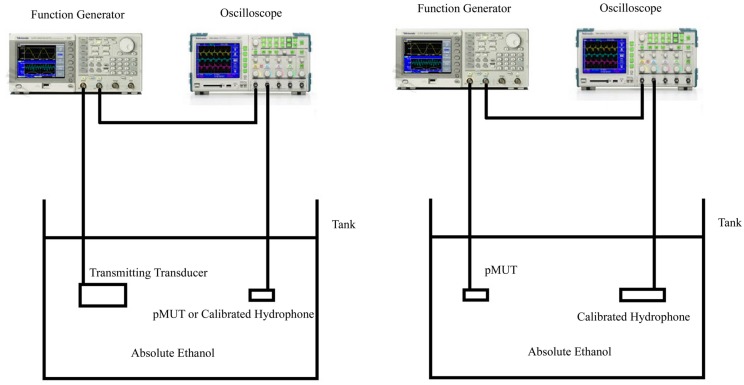
Receive (**left**) and transmit (**right**) sensitivity test of a pMUT.

The admittance curves of the pMUT were measured by a 4395A impedance phase analyzer (Agilent, Palo Alto, CA, USA). Figure 12 shows the admittance curves of pMUT with partially-etched piezoelectric film, where the resonant frequency in air is around 1.85 MHz. The measurements of both receive and transmit sensitivity were carried out in absolute ethanol. The receive sensitivity was measured by a comparison method as shown in Figure 13. The calibrated transducer (Figure 11c) in ten sine signals excitation transmits a sound wave at 1.79 MHz, and the pMUT and a calibrated hydrophone (Figure 11b) with 0.259 μV/Pa sensitivity were successively placed at the same position to receive the sound signal. The electric signals of hydrophone and pMUT were recorded by a TK DPO3012 oscilloscope (Tektronix, Beaverton, OR, USA).

**Figure 14:**
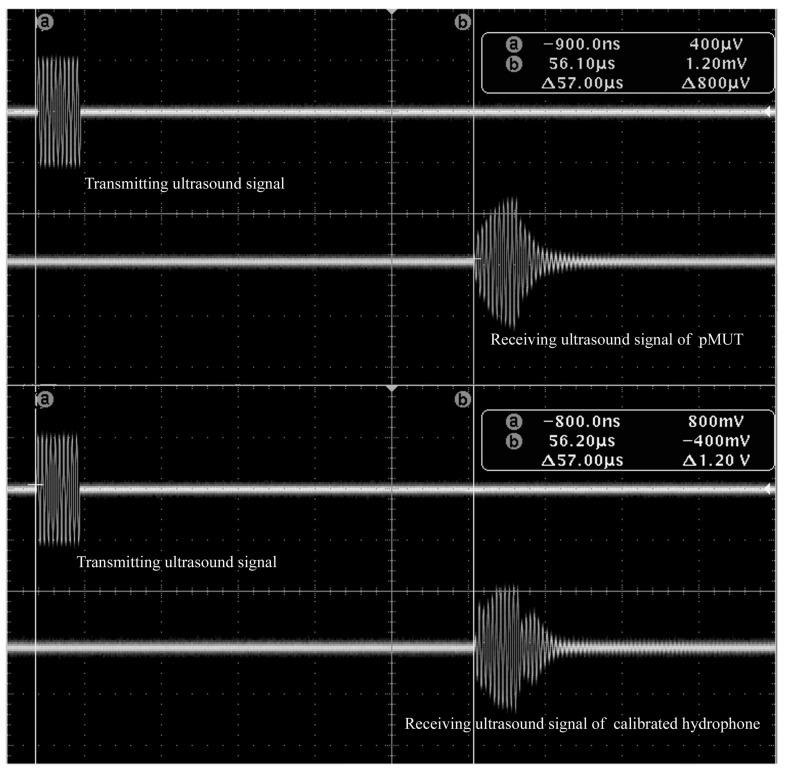
Measurement result of receiving sensitivity of a pMUT with partially-etched ZnO film.

The pMUT with partially-etched ZnO film was used as transmit transducer in the measurement of transmit sensitivity as shown in Figure 13. The excitation generated by the TK AFG 3012 function generator is ten sine signals at 1.79 MHz, and the peak to peak value is 10 V. A calibrated hydrophone with a sensitivity of 0.259 μV/Pa was placed at a certain distance from the pMUT, and the received sound signal was recorded by the TK DPO 3012 oscilloscope. Figure 14 shows the measured results of receive sensitivity of the pMUT with partially-etched ZnO, where the voltage amplitude of the pMUT and calibrated hydrophone are 9.9 mV and 2.12 mV, respectively, so the receive sensitivity of the pMUT is −238.35 dB (ref. 1 V/μPa), which is 3.05 dB higher than that with whole ZnO film.

**Figure 15:**
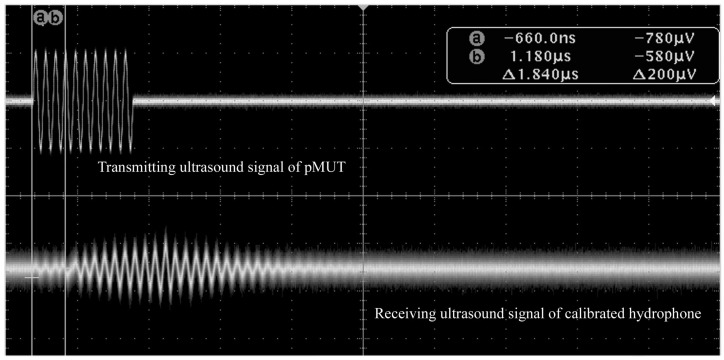
Measurement results of transmitting sensitivity of a pMUT with partially-etched ZnO film.

Figure 15 shows the measured transmit sensitivity results of the pMUT with partially-etched ZnO. After calibration, the measured transmit sensitivity of the pMUT element is 33.189 Pa/V, i.e., 150.42 dB (ref. 1 μPa/V) at 20 mm equivalent distance, which is 8.78 dB higher than that of pMUT with whole ZnO films. Besides the simulation results, the increase of receive and transmit sensitivity can also be attributed to the piezoelectric property diversity of ZnO film on different wafers and the fact that etching of a partial piezoelectric film can decrease the residual stress ignored in the FEM analysis. The receive and transmit sensitivity of the pMUT with partially-etched ZnO films are comparable to those based on PZT films that have better piezoelectric performance than ZnO [41], which indicates the structure of partially-etched piezoelectric films can significantly improve the sensitivity.

## 5. Conclusions

A square piezoelectric composite diaphragm of ZnO/Si/SiO_2_ was analyzed by the finite element method. The resonant frequencies are approximately equal by the finite element method and plate vibration theory, and the simulation results show that the resonant frequency of the pMUT is proportional to its thickness and inversely proportional to its width, respectively. The stress distribution results show the stresses in the centre and edge of the square piezoelectric composite diaphragm are bigger than in other areas, but they are opposite. Therefore, a centric electrode was designed to avoid neutralization of the stress in the centre and edges. In order to further improve the sensitivity, a pMUT with partially-etched ZnO film was adopted in which unnecessary ZnO film in the outer-ring was removed. The resonant frequency and sensitivities of three kinds of pMUTs including whole electrode, centric electrode and partially-etched ZnO film were analyzed by the finite element method (FEM). The receive sensitivity of the pMUT with partially-etched ZnO film is 3.3 dB and 6.8 dB higher than those with centric and whole electrodes, respectively, and the amplitude of a partially-etched ZnO film pMUT under a certain voltage is 5.5 dB and 30 dB higher than those with centric and whole electrode, respectively. The pMUT was fabricated by standard bulk micromachining fabrication techniques. The piezoelectric ZnO films were deposited by direct current magnetron sputtering and exhibit a densely packed structure with columnar crystallites preferentially oriented along the (002) plane. Both receive and transmit sensitivity were measured in absolute ethanol. The test results show that the removal of outer-ring ZnO films can raise the sensitivity notably. The receive sensitivity and transmit sensitivity of a pMUT with partially-etched ZnO film are separately −238.35 dB (ref. 1 V/μPa) and 150.42 dB (ref. 1 μPa/V), which are significantly higher than those with whole ZnO films.

## Figures and Tables

**Table 1:** Materials properties and thickness used in FEM simulation.

Materials	Young’s Modulus (GPa)	Density (kg·m^−3^)	Poisson’s Ratio	Thickness (μm)
ZnO	120	5.68	0.446	3
Si	167	2.33	0.28	6
